# Supplementary material for: pHmScarlet is a pH-sensitive red fluorescent protein to monitor exocytosis docking and fusion steps
Source: Nat Commun. 2021 Mar 3;12:1413. doi: 10.1038/s41467-021-21666-7 (PMC7930027; doi:10.1038/s41467-021-21666-7)
Supplement: Supplementary file 1 — Supplementary Information [file 41467_2021_21666_MOESM1_ESM.pdf]

Supplementary Figure 1

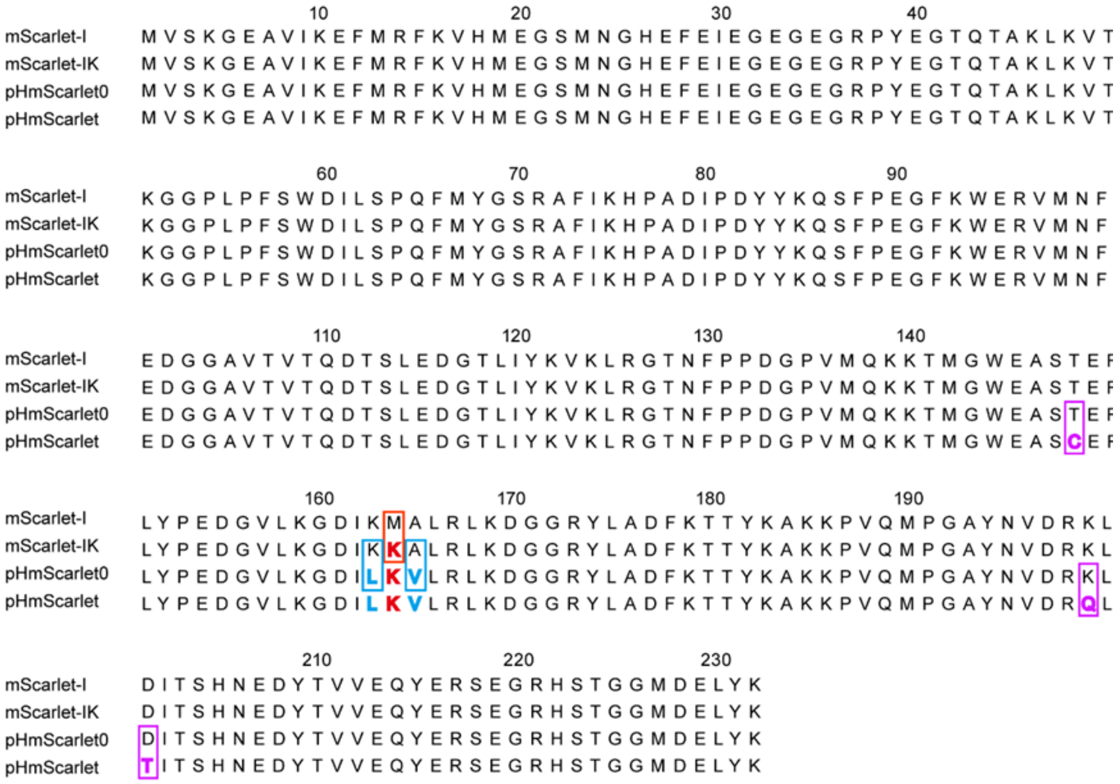

Supplementary Figure 1. Amino acid sequence alignment of mScarlet-I, mScarlet-IK, pHmScarlet0 and pHmScarlet. Red box indicated the mutation site from mScarlet-I to mScarlet-IK. Blue boxes represented the mutation residues from mScarlet-I to pHmScarlet0, and pink boxes showed the mutations from pHmScarlet0 to pHmScarlet.

Supplementary Figure 2

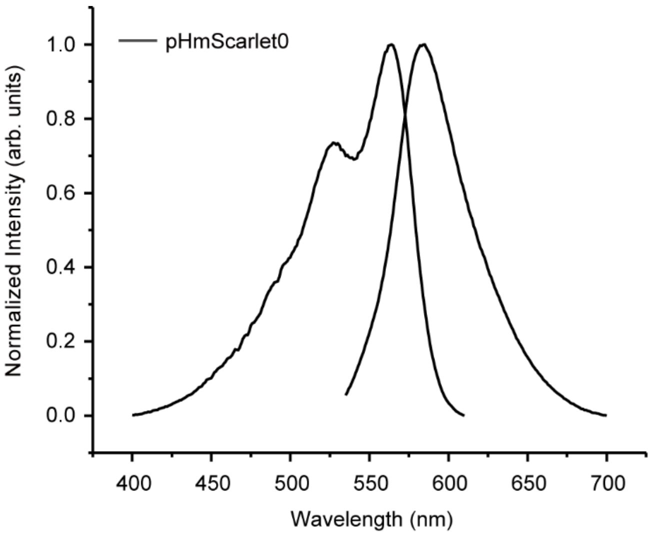

Supplementary Figure 2: Excitation and emission spectra of pHmScarlet0 at pH 7.4.

Supplementary Figure 3

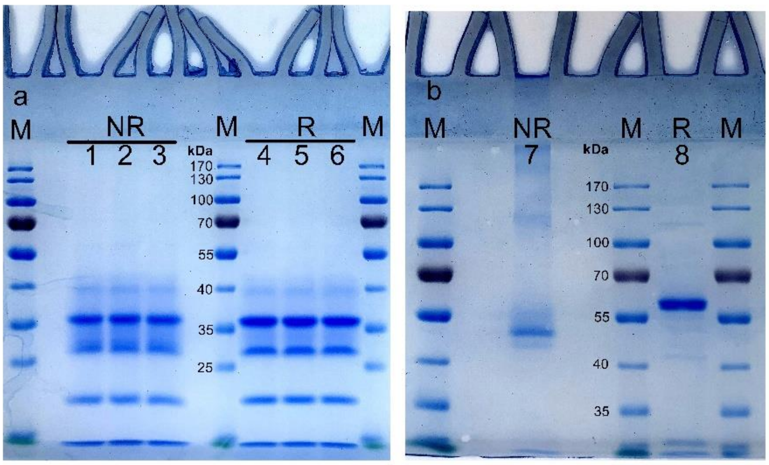

Supplementary Figure 3: Identification of the oligomeric states of pHmScarlet0 and pHmScarlet under oxidative and reducing environment. (a) purified mScarlet, pHmScarlet0, and pHmScarlet were analyzed by nonreducing SDS-10% PAGE under oxidative (without DTT/β-ME, line1, mScarlet; line2, pHmScarlet0 and line3, pHmScarlet) and reducing environment (with DTT/β-ME, line 4, mScarlet; line5, pHmScarlet0 and line 6, pHmScarlet). No significant difference was observed in the oxidative and reducing group for mScarlet, pHmScarlet0 and pHmScarlet, respectively. (b) Ero1α was included as a positive control. Purified Ero1α was loaded using buffer with or without DTT/β-ME and analyzed by nonreducing SDS-8% PAGE. Under oxidative environment, Ero1α migrated faster (lane 7) than that of under the reducing treatment (line 8). M: marker, NR: nonreducing, R: reducing. Gels were processed in parallel. Experiments were repeated two times.

Supplementary Figure 4

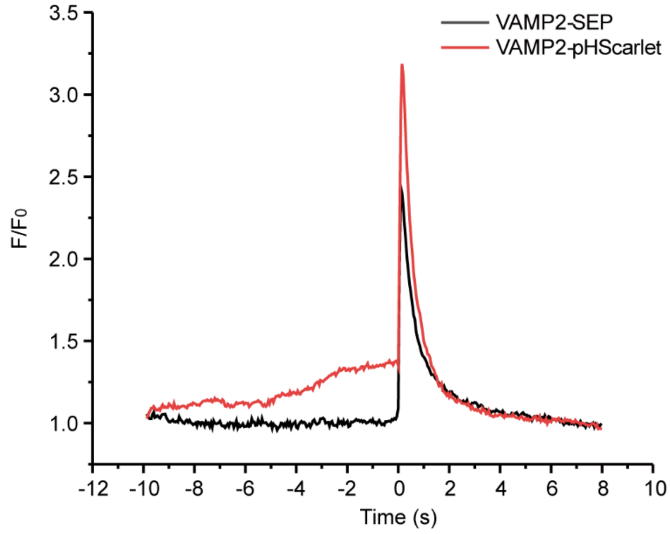

Supplementary Figure 4. Averaged normalized intensity traces of VAMP2-pHmScarlet (red) and VAMP2-SEP (black) vesicle exocytosis in response to high glucose and high [K+] stimulation in INS-1 cells. Mean fluorescence values for 108 events of SEP and 130 events of pHmScarlet labelling in six cells.

Supplementary Figure 5

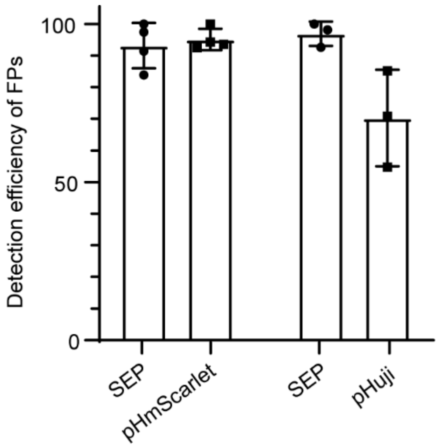

Supplementary Figure 5: Detection efficiency of vesicles using SEP, pHmScarlet and pHuji. Equal amount of plasmid DNA of SEP and pHmScarlet or SEP and pHuji were co-transfected into HT22 cells. The detection efficiency for different FPs is calculated by the number of fusion events detected by SEP, pHmScarlet or pHuji in one imaging channel over the total secretion events of two channels. Independent experiments were performed three times. The center values and the error bars in the graph represent mean value and error bars (SD).

Supplementary Figure 6

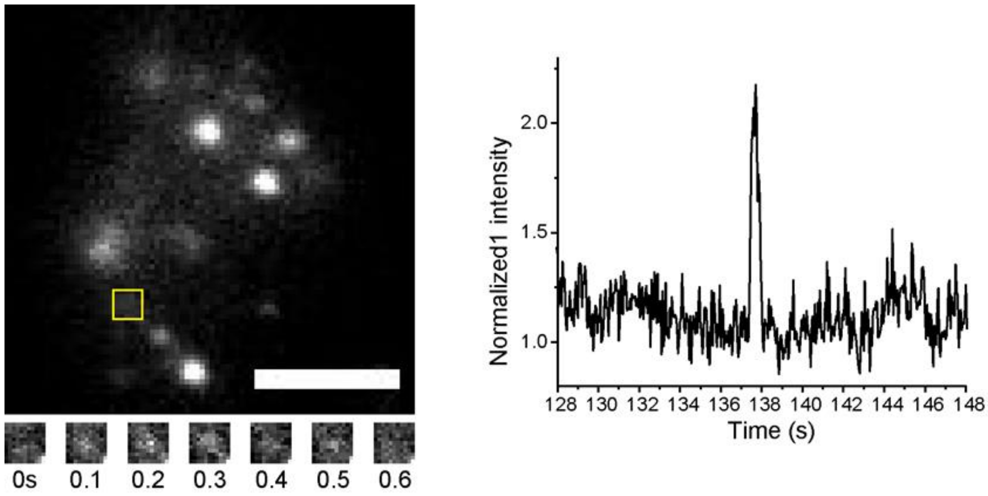

Supplementary Figure 6: Images of single-vesicle exocytosis in the soma region of primary mouse hippocampal pyramidal neurons expressing VAMP2-pHarlet. Fusion events in a single cell are indicated by yellow rectangles (left top), scale bars: 5 μm. Time-lapses of the marked exocytotic events are shown on the bottom (1 × 1 μm), scale bars: 1 μm; normalized intensity traces of the marked events are highlighted as red channels (right top).

Supplementary Figure 7

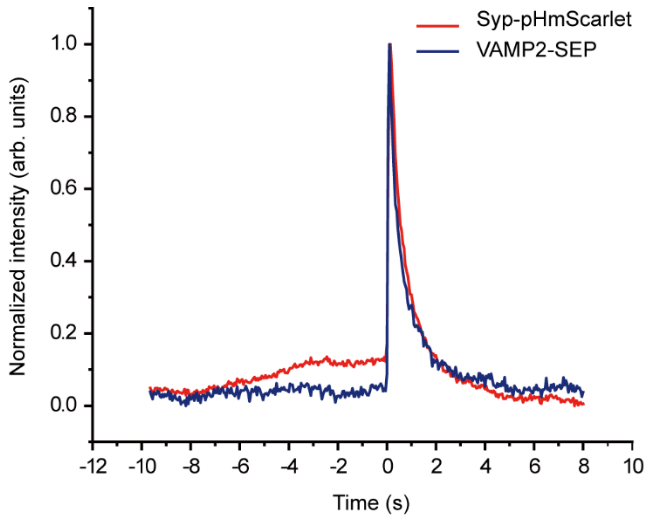

Supplementary Figure 7. Averaged normalized intensity traces of syp-pHmScarlet (red) and VAMP2-SEP (blue) vesicle exocytosis in response to high glucose and high [K+] stimulation in INS-1 cells. Mean fluorescence values for 102 events of SEP and 153 events of pHmScarlet labelling in six cells.

Supplementary Table 1

Fluorophore	Emission peak (nm)	Extinction coefficient ($10^3 M^{-1} cm^{-1}$)	QY (-)	Brightness ($10^3 M^{-1} cm^{-1}$)	Source
VO	581	90.9	0.40	36.36	Grimm et al. 2016
pHmScarlet0	590	86	0.48	41.53	
pHmScarlet	589	85	0.47	39.73	

Supplementary Table 1. In vitro brightness of red pH-sensitive fluorophores

Supplementary Table 2

Primer names	Sequences
pRSET-mScarlet-F	GATGGGGATCCATGGTGAGCAAGGGAGA
pRSET-mScarlet-R	TCGAATTCTTATTTATACAGCTCATCCATGCCTC
pRSET-SEP-F	ATGGGGATCCATGAGTAAAGGAGAAGAACT
pRSET-SEP-R	CTTCGAATTCTTATTATTTGTATAGTTCATCCATGC
pRSET-pHuji-F	GATCGATGGGGATCCATGGTGAGCAAGGGC
pRSET-pHuji-R	TTCGAATTCTTACTTGTACAGCTCGTCCAT
N1-H2B-F	AGATCCGCTAGCATGCCTGAACCGGCAAAA
N1-H2B-R	GCTTGAGCTCGAGCTTGGAGCTGGTGTACT
SNAP-F	CCACCGGTCGTTATGGACAAAGACTGCGAA
SNAP-R	AGTCGCGGCCGCTTTAACCCAGCCCAGGCT
H2B-mScarlet-F	AAGCTCGAGCTCAAGCTTGTGAGCAAGGGA
H2B-mScarlet-R	CCGCGGTACCGTCGACGATTTATACAGCTCATC
H2B-pHuji-F	GAGCTCAAGCTTGTGAGCAAGGGCGAG
H2B-pHuji-R	GCGGTACCGTCGACGACTTGTACAGCTCGTC
VAMP2-pHuji-F	GGGACCGGTGGAGTGAGCAAGGGCGAGGAG
VAMP2-pHuji-R	CGAGATCTTTACTTGTACAGCTCGTCCATGC
VAMP2-pHmscarlet-F	GGGGATCCCACCATGGTGAGCAAGGGAGAG
VAMP2-pHmscarlet-R	TCGCGGCCGCTTATTTATACAGCTCATCCA
syp-pHuji-F	CTAGCCACCATGGGAGTGAGCAAGGGCGAG
syp-pHuji-R	AGCTCCACCGGTTTACTTGTACAGCTCGTC
syp-pHmScarlet-F	CTAGCCACCATGGGAGTGAGCAAGGGAGAG
syp-pHmScarlet-R	AGCTCCACCGGTTTTATACAGCTCATCCATGC
OSER-pHmScarlet-F	CCACCGGTCGCCGTGAGCAAGGGAGAGGCC
OSER-pHmScarlet-R	TCGCGGCCGCTTTATTTATACAGCTCATCCATGCCTCC
Tubulin-pHmScarlet-F	CAGTCGACCGTGAGTGCATCTCCATCCA
Tubulin-pHmScarlet-R	GTGGATCCTTAGTATTCCTCTCCTTCTTCCTCACCC

Supplementary Table 2. Primer sequences used for constructions
