# Supplementary material for: pHmScarlet is a pH-sensitive red fluorescent protein to monitor exocytosis docking and fusion steps
Source: Nat Commun. 2021 Mar 3;12:1413. doi: 10.1038/s41467-021-21666-7 (PMC7930027; doi:10.1038/s41467-021-21666-7)
Supplement: Supplementary file 4 — Reporting Summary [file 41467_2021_21666_MOESM4_ESM.pdf]

nature research

Corresponding author(s): Lin Yuan; Pingyong Xu

Last updated by author(s): Feb 3, 2021

Reporting Summary

Nature Research wishes to improve the reproducibility of the work that we publish. This form provides structure for consistency and transparency in reporting. For further information on Nature Research policies, see our Editorial Policies and the Editorial Policy Checklist.

Statistics

For all statistical analyses, confirm that the following items are present in the figure legend, table legend, main text, or Methods section.

n/a	Confirmed	
☐	☒	The exact sample size (*n*) for each experimental group/condition, given as a discrete number and unit of measurement
☐	☒	A statement on whether measurements were taken from distinct samples or whether the same sample was measured repeatedly
☐	☒	The statistical test(s) used AND whether they are one- or two-sided<br>*Only common tests should be described solely by name; describe more complex techniques in the Methods section.*
☒	☐	A description of all covariates tested
☐	☒	A description of any assumptions or corrections, such as tests of normality and adjustment for multiple comparisons
☐	☒	A full description of the statistical parameters including central tendency (e.g. means) or other basic estimates (e.g. regression coefficient) AND variation (e.g. standard deviation) or associated estimates of uncertainty (e.g. confidence intervals)
☐	☒	For null hypothesis testing, the test statistic (e.g. *F*, *t*, *r*) with confidence intervals, effect sizes, degrees of freedom and *P* value noted<br>*Give P values as exact values whenever suitable.*
☒	☐	For Bayesian analysis, information on the choice of priors and Markov chain Monte Carlo settings
☒	☐	For hierarchical and complex designs, identification of the appropriate level for tests and full reporting of outcomes
☒	☐	Estimates of effect sizes (e.g. Cohen's *d*, Pearson's *r*), indicating how they were calculated

Our web collection on statistics for biologists contains articles on many of the points above.

Software and code

Policy information about availability of computer code

Data collection	Solis (Andor) was used for the TIRF image acquisition<br>"Laser control" plugin from Quick PALM 1.0.1 was used for the control of hardware in TIRF imaging<br>Hamamatsu was used for the SIM image acquisition
Data analysis	Image J<br>Origin 2017<br>Rolling algorithm

For manuscripts utilizing custom algorithms or software that are central to the research but not yet described in published literature, software must be made available to editors and reviewers. We strongly encourage code deposition in a community repository (e.g. GitHub). See the Nature Research guidelines for submitting code & software for further information.

Data

Policy information about availability of data

All manuscripts must include a data availability statement. This statement should provide the following information, where applicable:

- Accession codes, unique identifiers, or web links for publicly available datasets
- A list of figures that have associated raw data
- A description of any restrictions on data availability

No restrictions are placed on data availability. All source data and code are available upon reasonable request.

Field-specific reporting

Please select the one below that is the best fit for your research. If you are not sure, read the appropriate sections before making your selection.

☒ Life sciences ☐ Behavioural & social sciences ☐ Ecological, evolutionary & environmental sciences

For a reference copy of the document with all sections, see nature.com/documents/nr-reporting-summary-flat.pdf

Life sciences study design

All studies must disclose on these points even when the disclosure is negative.

Sample size	The sample size for each experiments has been specified in the Figure legends and supplementary information. To calculate the statistical significance between our probe and the controls both in vitro and in vivo, a minimal of 3 samples were used according to standard scientific conventions
Data exclusions	No data were excluded from the analysis
Replication	For all experiments, attempts at replication were successful
Randomization	For imaging and analysis, all samples were picked randomly from the slices
Blinding	No allocation into experimental group were performed

Reporting for specific materials, systems and methods

We require information from authors about some types of materials, experimental systems and methods used in many studies. Here, indicate whether each material, system or method listed is relevant to your study. If you are not sure if a list item applies to your research, read the appropriate section before selecting a response.

Materials & experimental systems

n/a	Involved in the study	
☒	☐	Antibodies
☐	☒	Eukaryotic cell lines
☒	☐	Palaeontology and archaeology
☐	☒	Animals and other organisms
☒	☐	Human research participants
☒	☐	Clinical data
☒	☐	Dual use research of concern

Methods

n/a	Involved in the study	
☒	☐	ChIP-seq
☒	☐	Flow cytometry
☒	☐	MRI-based neuroimaging

Eukaryotic cell lines

Policy information about cell lines

Cell line source(s)	INS-I cell, Human Bone Osteosarcoma Epithelial (U-2 OS) cell and mouse Hippocampal Neuronal Cell Line HT-22 from laboratory
Authentication	The cell line has been authenticated
Mycoplasma contamination	The cell line tested negative for mycoplasma contamination
Commonly misidentified lines (See ICLAC register)	No commonly misidentified cell lines were used in this study

Animals and other organisms

Policy information about studies involving animals; ARRIVE guidelines recommended for reporting animal research

Laboratory animals	P1–2 day old Sprague Dawley rat pups
Wild animals	No wild animals were used in this study
Field-collected samples	Rat pups were placed individually into a clean cup in a humidity-controlled (about 50%) cage with maintained temperature (about 30 °C) to maintain the rat pups at a normal body temperature.

Ethics oversight	All animal experiments were approved by and performed in accordance with the guidelines of the Animal Care and Use Committee of Institute of Genetics and Developmental Biology, Chinese Academy of Sciences (Approval code: AP2013003 and AP2015002).

Note that full information on the approval of the study protocol must also be provided in the manuscript.
