# Supplementary material for: pHmScarlet is a pH-sensitive red fluorescent protein to monitor exocytosis docking and fusion steps
Source: Nat Commun. 2021 Mar 3;12:1413. doi: 10.1038/s41467-021-21666-7 (PMC7930027; doi:10.1038/s41467-021-21666-7)
Supplement: Supplementary file 6 — Description of Additional Supplementary Files [file 41467_2021_21666_MOESM6_ESM.pdf]

Description of Additional Supplementary Files

**Title:** Supplementary Movie 1
**Description:** Continuous imaging of vesicle fusion labeled by VAMP2-pHmSarlet in an INS-1 cell corresponding to Fig. 4a.

**Title:** Supplementary Movie 2
**Description:** Continuous imaging of vesicle fusion labeled by VAMP2-pHmSarlet in a HT-22 mouse hippocampal neuronal cell corresponding to Fig. 4h.
